# Visual hallucinations in dementia with Lewy bodies originate from necrosis of characteristic neurons and connections in three-module perception model

**DOI:** 10.1038/s41598-022-18313-6

**Published:** 2022-08-19

**Authors:** Shigetoshi Nara, Hiroshi Fujii, Hiromichi Tsukada, Ichiro Tsuda

**Affiliations:** 1 Graduate School of Natural Science & Technology, Okayama University, Okayama, 700-8530 Japan; 2 Faculty of Information Science and Engineering, Kyoto Sangyo University, Kyoto, 603-8555 Japan; 3 Center for Mathematical Science and Artificial Intelligence/Chubu University Academy of Emerging Sciences, Chubu University, Aichi, 487-8501 Japan; 4 Chubu University Academy of Emerging Sciences/Center for Mathematical Science and Artificial Intelligence, Chubu University, Aichi, 487-8501 Japan

**Keywords:** Neuroscience, Diseases, Neurology, Pathogenesis, Signs and symptoms, Mathematics and computing

## Abstract

Mathematical and computational approaches were used to investigate dementia with Lewy bodies (DLB), in which recurrent complex visual hallucinations (RCVH) is a very characteristic symptom. Beginning with interpretative analyses of pathological symptoms of patients with RCVH-DLB in comparison with the veridical perceptions of normal subjects, we constructed a three-module scenario concerning function giving rise to perception. The three modules were the visual input module, the memory module, and the perceiving module. Each module interacts with the others, and veridical perceptions were regarded as a certain convergence to one of the perceiving attractors sustained by self-consistent collective fields among the modules. Once a rather large but inhomogeneously distributed area of necrotic neurons and dysfunctional synaptic connections developed due to network disease, causing irreversible damage, then bottom-up information from the input module to both the memory and perceiving modules were severely impaired. These changes made the collective fields unstable and caused transient emergence of mismatched perceiving attractors. This may account for the reason why DLB patients see things that are not there. With the use of our computational model and experiments, the scenario was recreated with complex bifurcation phenomena associated with the destabilization of collective field dynamics in very high-dimensional state space.

## Introduction

**Figure 1:**
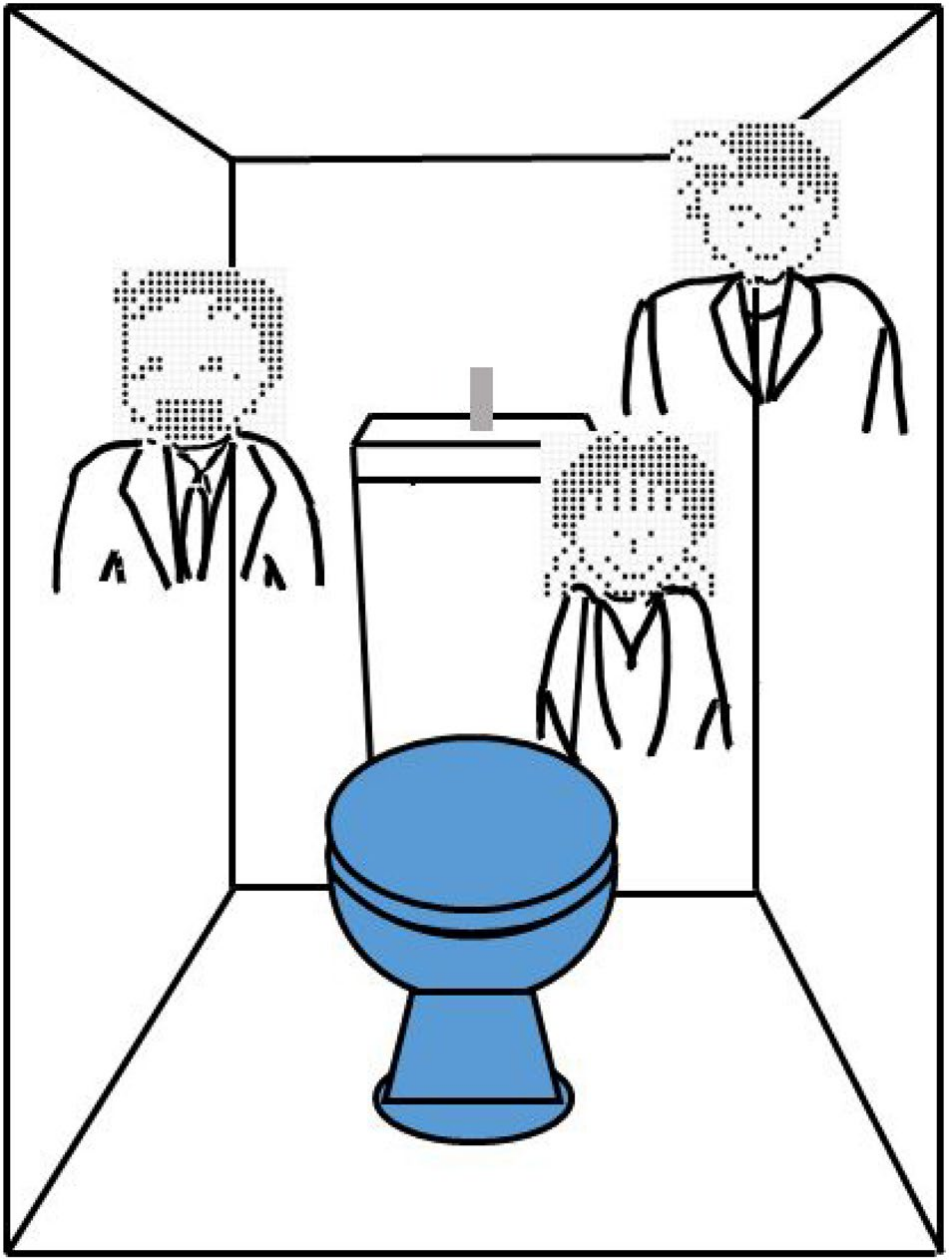
An illustration of a hallucinatory image drawn by one of the authors (S.N.) based on the oral description by a patient with RCVH-DLB who said as follows in an interview for a TV educational program of Japanese broadcast station. The patient said “strangers are in the bathroom, so I am scared and cannot enter the room”.

For several decades, dementia with Lewy bodies (DLB hereafter) has been an area of interest for many people in medicine, neurology, pharmacology, psychiatry, and brain sciences research. One of the reasons for this interest is that, in addition to typical symptoms of dementia, DLB patients exhibit a very peculiar symptom called “recurrent complex visual hallucinations (RCVH)” with high probability at rather early or intermediate stages of pathological progression. A very characteristic feature of RCVH-DLB is that patients with DLB see things that are not there. Moreover, the subject of the hallucinations is not inconsistent with environmental situations surrounding the patients; for example, patients see persons, animals, insects, flowers, and so on. The majority of patients manage to communicate with family members or doctors regarding such hallucinations and are able to describe their hallucinatory images. Figure 1 shows an illustration of a hallucinatory image drawn by one of the authors (S.N.) based on the oral description by a patient with RCVH-DLB, who said “strangers are in the bathroom, so I am scared and cannot enter the room”. Let us note that Fig. 1 is an *imaginary illustration* with use of the oral description of hallucination told by a patient. Readers are able to get more accurate medical data of symptoms in the references (see^1,2^ shown later). However, aside from the medical symptom data, to investigate the information processing supposed to occur in patients’ brain, objective methods to visualize the hallucinatory image may be necessary.

A large number of papers have been published, even when only considering the rather theoretical research, but searching all of these papers is nearly impossible. In noteworthy papers for the authors^1–10^, one of the typical reviews that investigated pathological data and considered their phenomenological aspects was published in Behav. & Brain Sci. This paper emphasized the pharmacological aspects concerning neuromodulator impairments in the cortex (see^1^). Collerton et.al investigated a large number of pathological cases of visual hallucinations, and after careful consideration regarding various kinds of models reported before 2005, they proposed their model, which they called the “Perception and Attention Deficit model (PAD)”; however, the essential understanding of RCVH is still obscured by the brain’s hypersuperior complexities.

In the successive review paper published in J. Conscious. Stud., (see^2^), various data published during the decade following the previous review are shown and detailed considerations are given. The main points of this review focused on neuro-psychological aspects but incorporated novel trials, as much as possible, with theoretical viewpoints based on mathematical sciences and nonlinear physics. Below, we summarize several important points of their review, which were necessary for us to construct our computational model proposed in the present paper.

1. Measurement technologies to observe biological cells, systems, and organs from the microscopic to macroscopic levels have advanced greatly in the past decade. These tools enable us to obtain biophysical, biochemical, physiological, pharmacological data with finer space-time resolution. For example, it has become possible to record the activities of neural networks on a very large spatiotemporal scale by noninvasive methods, such as diffusion-tensor imaging of magnetic resonance (DTI-MR), functional MR imaging (fMRI), positron emission tomography (PET), single-photon emission computed tomography (SPECT), etc. Essentially, these techniques result in the situations to request researchers to analyze large amounts of data, called “big data”, in computer science & technology^11–20^.
2. Despite the substantial developments in noninvasive technologies that allow individuals to observe neural activities, a detailed understanding of the functional roles of neuromodulators (transmitters) remains quite insufficient. A large number of papers have discussed the effects of pharmacological interventions on DLB and many brain diseases as well; however, most researchers have difficulties in getting quantitative data because it is impossible to expose patients with brain diseases to arbitrary administration of neuromodulators, such as acetylcholine and dopamine, as medical treatments. Instead, a large number of experiments have been conducted in the brains of animals, such as rats or mice; however, obtaining quantitative data about the influence of these treatments on advanced functions is quite difficult in such animals.
3. RCVH may be attributed to dynamic disorders distributed rather widely among visual perceptual systems and is typically considered to be a network disease, as suggested by ffytche^5^. These disorders originate not only from neuronal atrophy and dysfunction of synaptic connections in several separate fields caused by aggregated Lewy bodies (LBs) but also from impairment of neuromodulator release over widely entangled regions of axons and dendrites. It may participate in destabilizing interactive dynamics among several fields, per se, normal release works to maintain functional self-consistency in veridical perceptions, particularly in visual fields of the occipital cortex and frontal cortex, including neural links among these two regions and the temporal cortex.

Therefore, although various forms of anatomical and functional deterioration in the visual perception network in DLB patients have been discovered via a large number of measurements with the use of advanced technologies, as noted above, there is insufficient knowledge concerning the neuropathological origins of RCVH in patients with DLB. More specifically, widely distributed necrotic neurons in complex neural networks has prevented us from specifying these origins. Thus, computational modeling provides a meaningful, systematic method to investigate the primary effects of neuron necrosis in the visual system since there is considerable uncertainty in measured data even when they are obtained with modern observation technologies.

## Computational model of visual perception and destabilization of perceiving attractors

### Perceiving attractors as an interpretative scenario of veridical perception

Considering the factors stated in the previous section, we suggest an improved scenario of visual perception processes mainly based on the viewpoints of mathematical science and nonlinear-nonequilibrium physics. This scenario would respect the suggestion of Marr^21^, in which the three stages that one should go through to understand information-processing task are suggested, i.e., computational theory $\Rightarrow$ algorithmic representation $\Rightarrow$ hardware implementation (experiment). Therefore, in the first stage, the idea of perceiving attractors to represent convergence of neural processing to veridical visual perceptions is evoked. Such computational ideas and approaches based on the concept of an attractor framework have been used in many works since the Hopfield model was developed^22^. A typical treatment is shown in^6^.

Our computational idea includes the following characteristic features, which are presented in a list to allow us to emphasize the important points.

- It is well known that the visual information stream from the occipital cortex takes two main paths: the superior (dorsal) longitudinal pathway^23,24^ and the inferior (ventral) longitudinal pathway^25,26^. Association fibers to support the former stream are generally called the “superior longitudinal fasciculus”, and the latter are called the “inferior longitudinal fasciculus”.
- Once input is transmitted to the visual cortex, “bottom-up information” is generated in the visual cortex and sent to both the frontal cortex and temporal cortex via the two aforementioned pathways, including visual information processing performed by occipital and peripheral cortexes.
- Responding to this input, the frontal and temporal cortex interact with each other via the hippocampal region. The frontal cortex generates a perceptual prior, the concept of which was first proposed by one of the authors (I. Tsuda)^27^. The temporal cortex recalls parts of memories, one by one, that contain bottom-up information and simultaneously forms “scene (context) candidates” in subconscious state, which may correspond to the concept of active inference introduced by Friston et al.^4,28^.
- Interactions between the temporal and frontal cortices produce two types of “top-down information” that are then sent to the visual cortex. The first is from the temporal cortex and compensates or even overcompensates insufficiencies, when there is inadequate bottom-up information, to enable for individuals to recall correct parts and scene (context) in visual inputs. The second is from the frontal cortex and includes attention in arousal consciousness; this information is intended to generate updated visual information to eliminate or at least decrease errors.
- In visual perceptions, on the other hand, it has been generally accepted that a few neuromodulators, such as acetylcholine, are critically important in advanced functions of the human brain. It is also accepted that cholinergic cells exist in the basal ganglia; in particular, the nucleus basalis of Meynert (nbM) elongates long axon fibers to very wide fields of the neocortex from the frontal lobe to even the occipital lobe^1,2^. Though pertinent mechanisms have not yet been fully clarified, it has at least been verified that acetylcholine released by cholinergic cells regulates activity levels of advanced functions, for instance, attention, arousal, intention, etc.
- According to two top-down pieces of information transmitted to the visual cortex, new bottom-up information is generated and is sent to the temporal & frontal cortices. These processes are recurrently repeated until veridical perception is achieved and accepted by one’s consciousness in the arousal state.

**Figure 2:**
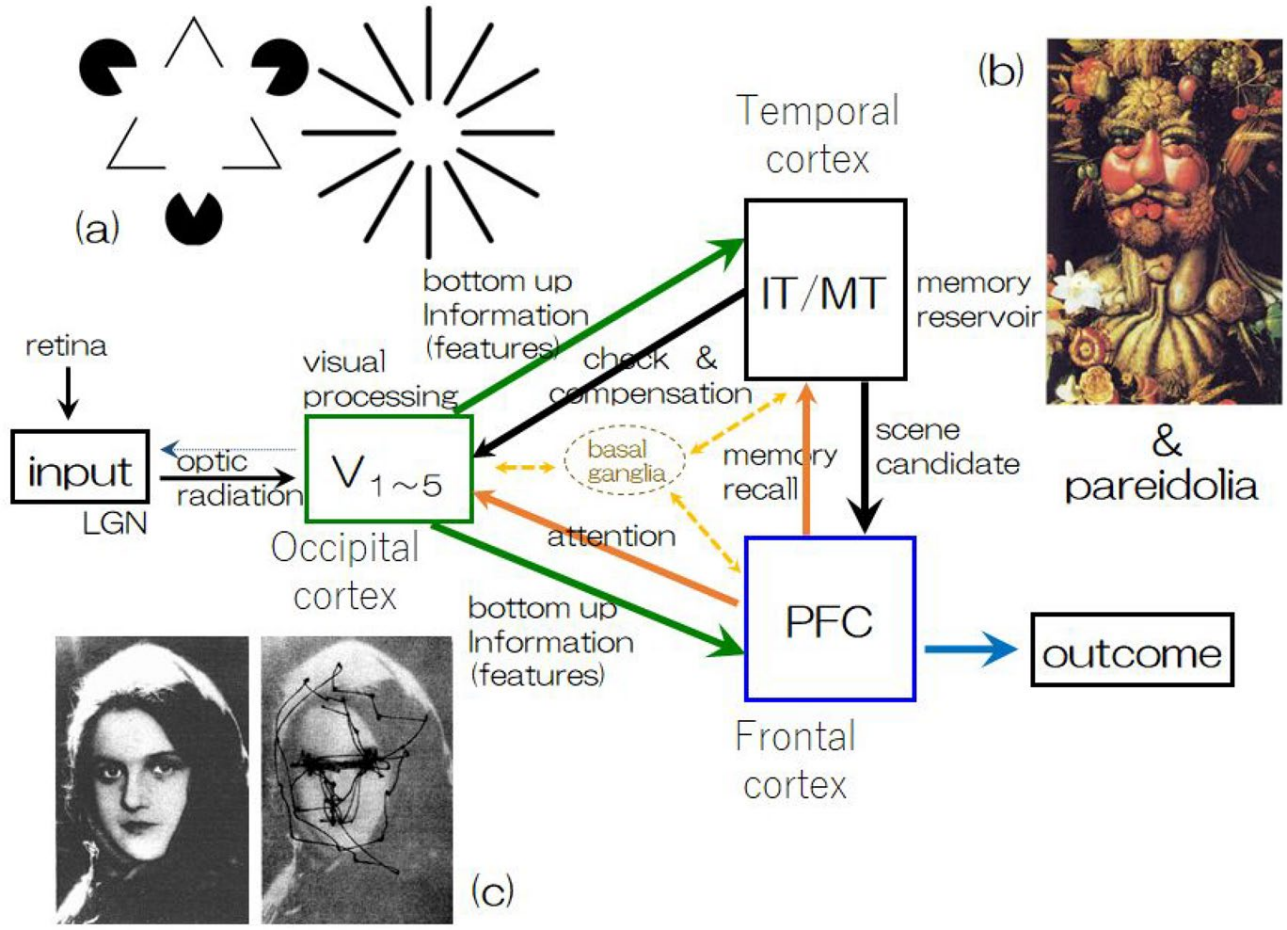
A three-module model of visual processing that enables veridical perception. (**a**) A well-known illusion that shows a representative example of compensation resulting from the subconscious interaction between the visual cortex and temporal cortex. (**b**) A figure painted by Giuseppe Arcimboldo in 1591 called *Vertumnus, porträttet av Rudolf II*, which induces a typical subconscious function of memory recall of parts, one by one (vegetable & fruits, etc.), and empirical imaging of the entire scene or context in the mind. Portions were omitted to show image examples of “pareidolia”. (**c**) A typical example of attentional eye movement reported by A. L. Yarbus (1967) driven by top-down information transmitted from the frontal cortex. The basal ganglia regulate and adjust the functional activities of all fields (cortices) by means of fine control of the inhomogeneous release of neuromodulators (transmitters).

These considerations lead us to a three-module model of visual perception and an interpretative scenario of veridical visual perceptions, as shown in Fig. 2, which we call a “perceiving attractor”. After inputs to the visual cortex are provided, neural activity in the three regions-the visual, temporal, and frontal cortices-reaches a certain converged state and results in veridical perception produced by the frontal cortex. Via this process, a normal subject is able to recognize a specific person quite easily from incoming visual inputs containing abundant image data, for example, many photographs and many scenes. This recognition function is one of the typical examples of veridical perception; in other words, one forms concepts with respect to the specific person. Mathematically speaking, this function is regarded as embedding many-to-one correspondence mapping into the state space of neural cooperative activity in several fields of the brain, whereas the activity is represented as rather low-dimensional (weak) chaos or may be regarded as a limit cycle^29^. Given these considerations, it is possible to introduce a hypothesis that such low-dimensional weak chaos in neural activity contributes to resulting in veridical perception and corresponds to perceiving attractors in a huge dimensional space of neural activity. Moreover, in our model, this weak chaotic attractor is approximated to be a new type of attractor, in which both the limit cycle and fixed pattern are incorporated together into one attractor, as shown in later computer experiments.

Finally, it should be noted that in the present model, we suggest that detailed information such as context^30^ or attention^1^, which have been considered in previous works, are included in collective fields, where they are not explicitly examined but may be encompassed in the collective field to generate hodological functions introduced by ffytche^5^.

### Destabilization phenomena of perceiving attractors corresponding to RCVH in DLB

It is generally accepted that many brain diseases are attributed to large unrecoverable necrosis of neurons and synaptic connections over widespread regions of the brain. Depending on the inhomogeneous distribution of such necrosis, many typical features of pathological neuron-network activities corresponding to each brain disease have been observed. MRI, including more advanced techniques, such as fMRI, and DTI-MR, are appropriate methods to detect differences in anatomical and pathological data between a brain with a disease and a normal brain.

So, let us start with a comparison of neuropathological and/or neuroanatomical properties between normal subjects and patients with DLB, then we introduce these properties into our model as factors that destabilize perceiving attractors due to necrosis of neural cells and synaptic connections or due to a neuromodulator releasing disorder. Various measured data were roughly classified into the following four kinds of atrophy data.

1. Volume loss of Gray matter observed by means of voxel-based morphometry (VBM) or region of interest (ROI) analysis of MRI scans.
2. White matter (association fibers) atrophy detected by MRI and DTI-MR.
3. PET, SPECT, and fMRI measurements to inspect hypometabolism and hypoperfusion in the brain.
4. Subcortical degeneration observed by means of MRI, SPECT, etc.

Each topic has been investigated by many researchers, for instance in the review of Watson in detail^31^, but let us pick up several important points that support our consideration stated later. First, with respect to (1), MRI data indicate that gray matter loss in the brains of DLB patients occurs over the entire region of the brain in comparison with that in normal subjects, *although atrophy of the temporal lobe is not yet so severe in DLB patients at the initial and/or intermediate stages of the disease.* However, the data are not quantitatively accurate because there is a rather wide distribution of data among individual patients. For instance, a few papers^32–34^ report the rates of cerebral atrophy, but data vary widely, and it is difficult to specify a particular degree of neuronal necrosis. Additionally, Firbank et al.^35^ employed the following expression: “DLB: less hippocampal atrophy in CA1 and subiculum compared to AD”, which shows that it is difficult to quantify the degree of necrosis among neurons corresponding to DLB in our computer experiments.

Regarding (2), two methods are used to obtain data, i.e., white matter hyperintensity (WMH) measurements in MRI, fractional anisotropy (FA) and mean diffusivity (MD) measurements in DTI-MR. There is a wide variety of WMH data reported by many researchers also, and a representative statement is given as “As expected, there was a trend toward less WMH in the control group when compared with AD and DLB”^31^. Such a situation is similar with respect to measurements of FA and MD. Data are described as “decreased FA” or “increased MD” in comparison with the measurements of normal subjects. Furthermore, there is a great difficulty in evaluating DTI-MR data because DTI scans do not indicate the direction of signal propagation (inward or outward). DTI scans only show axon connections between two regions in gray matter since the measured diffusion constant data indicate only the anisotropic diffusion direction of water molecules in three dimensions. Thus, although it is difficult to find the quantitative data necessary to accurately take in neuronal necrosis and synaptic connections disorder in our computer experiments, these data^11–17,31–40^ are interpreted as described below and can be employed in our experiments as *parameter-dependent factors*.

- Atrophy *does exist* over the entire region of the brain but relatively little atrophy of the temporal lobes is present in patients in comparison with that in normal subjects.
- Reduced FA and increased MD are clearly observed in the region of the inferior longitudinal fasciculus (occipital-temporal connection) in patients in comparison with that in normal subjects.
- Additionally, reduced FA and increased MD are clearly observed in the region of the superior longitudinal fasciculus (occipital-parietal-frontal connection) in patients in comparison with that in normal subjects.

Concerning (3), signal imaging of PET and SPECT revealed hypometabolism and hypoperfusion in the occipital cortex^39,40^, which suggests considerable necrosis of neurons and surrounding synaptic connections. It should be noted that the authors included disorders of the optic radiation in the posterior thalamic area that were observed on DTI-MR scans, in which the main stream of visual information is transferred from the lateral geniculate nucleus (LGN).

Regarding (4), degeneration of cholinergic cells (particularly nbM) and dopaminergic cells (particularly striatal cells)^36^ have been detected in DLB patients using MRI and SPECT. There are many neuromodulators that influence visual information processing and various advanced functions in the brain. Many publications have examined acetylcholine from the molecular and pharmacological perspectives, e.g., nicotinic acetylcholine receptors^38^. However, most of the papers related to mental diseases are about global trends of the association between the amount of acetylcholine in the brain and behavioral observations among humans and rodents. It is quite difficult to determine the causal links of the concentration of acetylcholine in the brain with a quantitative evaluation of the performance of advanced functioning (e.g., attention, arousal), the pathological symptoms of DLB and hallucinations. Many studies have examined impairments of acetylcholine-releasing cell-groups in the brain stem, but the detailed mechanism how to influence DLB and/or hallucinations has not yet been clarified. However, activity over a rather wide field can be regulated by the controlled release of acetylcholine. Therefore, in the present paper, we introduce a threshold fluctuation in each module as a symbolic effect of acetylcholine release impairment.

Therefore, the situations stated above indicate that a parameter-surveying investigation using a computational model concerning neuronal necrosis and synaptic connections is meaningful to investigate what and how large deficits in neural activity due to degeneration of neural cells in the occipital lobe (visual fields). This approach would also be useful for investigating the effects of substantial atrophy of neural connections between occipital-temporal and occipital-frontal lobes^5,12^. Moreover, this technique could be used to shed light on the issues observed in the transmission of information during visual processing that leads to perception.

Now, these observed results and their considerations bring us the following interpretative scenario of RCVH-DLB within the framework shown in Fig. 2. First, there was considerable atrophy in the input (visual) module. There was relatively less atrophy in the memory module and perceiving module. Second, bottom-up information from the input module to both the memory and perceiving modules included serious deficits; therefore, both modules were insufficient to use to construct plausible representations of prior perception in the perceiving module and plausible representations of context prior in the memory module. Third, attention information sent to the input module from the perceiving module (top-down information) was prevented by degeneration of the superior longitudinal fasciculus and surrounding connections, which results in attention deficits^41^. Additionally, compensation by the memory module to the input module (the other top-down information provided) that was achieved subconsciously was hindered due to damage to the inferior longitudinal fasciculus, which could cause overcompensation. Fourth, in these pathological environments, prior perception generated by the perceiving module and constructed context in the memory module affected by overcompensation could become inconsistent and interact via the uncinate fasciculus. Neither inevitably nor coincidentally, the uncinate fasciculus connecting perceiving module and memory module was essentially intact in patients with DLB in the present observation at rather initial or intermediate stages of pathological progression. It should be noted that the three authors in the present paper (see^30^) discussed RCVH and emphasized the primary role of dysfunction in the inferior longitudinal fasciculus in this aspect of the disease.

A large contradiction between two previously evoked perceptions due to insufficient input information (bottom-up information) results in a lack of self-consistency between two top-down pieces of information generated in the memory module and perceiving module. This inconsistency causes the collective fields that are mutually exchanged in the three modules to be unstable. Then, this instability destabilizes a relevant perceiving attractor; this result may be called critical fluctuation (or precursor phenomena) as it is associated with “phase transition from normal to dementia”. Here, we do not get involved so deeply in analogy between phase transitions in physics and disease appearance (progression), nor in actual applications to detection of predisease symptoms^42^. Thus, let us only consider the conceptual similarity to phase transitions in the present paper. Hence, the unstable fluctuation of the collective field may give rise to transient emergence of mismatched perceiving attractors; in other words, hallucinations occur. The above consideration reveals that RCVH occurs when the dysfunction of the visual processing system, which encompasses various regions in the brain, reaches a critical stage of destabilization due to characteristic necrosis of neurons and their synaptic connections. Furthermore, the damage inflicted by LBs may be delicately balanced in several areas in the neocortex, thus causing critical fluctuations in collective fields between veridical perception and hallucinatory perception. This means that a certain systematic combination of partially lesioned fields is critical in the occurrence of RCVH. This type of destabilization may be rare in individuals with brain diseases but its presence has been associated with a notable number of pathological symptoms, particularly in patients with DLB at the initial and/or intermediate stages of disease.

Generally, the destabilization of stationary (attractor) dynamics and critical fluctuations associated with destabilization have been investigated and characterized by finding bifurcation phenomena in the concerned system. One of the very difficult problems in the present case is the extremely high-dimensional state space that must be addressed even if we drastically simplify the visual perception system and employ the three-module model stated above. Hence, there are many characteristic parameters that cause instability; in the present case, the number of dead neurons due to necrosis brought about by disease and the configurational positions of these neurons in the state space are also parameters that change dynamical instability. Three modules in the present system give large varieties in selections of key parameters. Furthermore, there is a large variety of synaptic connections between each pair of modules with respect to the number of surviving connections and their configurations that were not affected by necrosis caused by disease. However, in the highly diverse bifurcation phenomena in our computer experiments, we managed to find a consequential number of critical fluctuations in perception corresponding to hallucination-like instabilities in trials of primary parameter selection considering the data acquired in recent measurements.

In the next section, the results of computer experiments are reported, although they do not yet cover all possible selections of primary parameters.

## Methods

### Computer experiments to implement perceiving attractors in a mathematical model of veridical perceptions

Following the idea and the scenario presented in the previous sections, we constructed a computational model and implemented computer experiments. The important points in the practical methods are described as follows.

1. Employment of a binary-state neuron model and discrete time scheme regarding state dynamics of neuron network. The neuronal activity of this system is represented by a high-dimensional state vector ${{\varvec{s}}}$.
2. The number of neuron elements is $N_V$ for the visual module, $N_M$ for the memory module, and $N_P$ for the perceiving module; hence, the total number of elements is $N=N_V + N_M + N_P$. In the present experiments, considering computational performance, $N_V = 900$, $N_M = 1200$, and $N_P = 900$; therefore, the total number of elements is $N = 3000$.
3. As an example of perceiving attractors, *K* limit cycle attractors were employed, where each cycle attractor contained periodic *L* step sequences of state vectors per cycle in visual module and memory module. In contrast, *L*-sequences of one fixed state vector were embedded in the perceiving module, which is metaphor of a specific perception, during *L*-steps of cyclic change in visual and memory modules, as shown in the subsequent figures. $K=11, \;\; L=10$ were employed in the present experiments based on the authors’ experiences when utilizing weak chaos, shown in previous papers, to solve ill-posed problems, such as memory search, certain robotic movements, etc., in which rather longer periods of limit cycle attractors lead to better functional performances. Here, we introduce only one reference^43^ instead of omitting the detailed explanations about other studies.

Now, for a simplicity of description, let us begin with updating rule of the state vector in a single module, which is defined as

1 $$\begin{aligned} s_{i}(t+1) = \mathrm{sgn} \left( \sum _{j=1}^N \varepsilon _{ij} w_{ij} s_{j}(t) \right), \mathrm{sgn}(u) = \left\{ \begin{array}{cc} 1 & \;\;(\mathrm{if}~\;u \ge 0)\\ -1 & \;\;(\mathrm{if}~\; u < 0) \end{array} \right. \end{aligned}$$

where each $s_i(t)$ takes discrete values $s_i=\pm 1$, and *N* is the total number of neural elements. $s_i= +1$, and $s_i= -1$ represent “active”, and “non-active” of the *i*-th neuron, respectively. The state of the system at the time *t* is represented by a *N*-dimensional state vector ${{\varvec{s}}}(t)$. $\mathrm{sgn}(u)$ is the sign function and $\{w_{ij}\}$ is a synaptic connection matrix. $\{\epsilon _{ij}\}$ is a matrix of binary values $\epsilon _{ij} =1$ or 0, with $\sum _j \varepsilon _{ij}=r^I_i$ and $\sum _i \varepsilon _{ij}=r^O_j$, where the connectivity parameter $r^{I(O)}_ i\; (0 \le r^I_i, r^O_i \le N)$ is the number of fan-in and fan-out connection number. Hence, $r^I_i$ and $r^O_i$ represent surviving pre- and post-synaptic connections that were not damaged by necrosis due to disease. If we set $r^I_i = r^O_i = 0$ for a specified neuron *i*, then it means that the *i*-th neuron is interpreted as dead because no input can be received and no outcome is given.

Employing the pseudo-inverse method (orthogonalized learning method)^44,45^, the synaptic connection matrix of cycle attractors is given by

2 $$\begin{aligned} w_{ij} = \sum _{\mu =1}^{K}\sum _{\lambda =1}^{L}\xi _{i}^{\mu ,\lambda +1} ~^\dagger \xi _{j}^{\mu ,\lambda }, ^\dagger \xi _{j}^{\mu ,\lambda } = \sum _{\alpha }^{K}\sum _{\beta }^{L} (o^{-1})_{\alpha ,\beta }^{\mu ,\lambda }\xi _j^{\alpha ,\beta }, o_{\alpha ,\beta }^{\mu ,\lambda } = \sum _j\xi _j^{\mu ,\lambda }\xi _j^{\alpha ,\beta }, \end{aligned}$$

where $\{ {{\varvec{\xi }}}^{\mu ,\lambda } = (\xi _1^{\mu ,\lambda }, \cdots , \xi _i^{\mu ,\lambda }, \cdots , \xi _N^{\mu ,\lambda }) \; | \; \xi _i^{\mu ,\lambda } = \pm 1, \; \mu =1, \cdots K, \; \lambda =1, \cdots , L \}$ is a set of attractor states (*N* dimensional state vectors) and we employ the condition ${{\varvec{\xi }}}^{\mu ,L+1} = {{\varvec{\xi }}}^{\mu ,1}$. *K* and *L* are the number of cycles and the period of each cycle, respectively. $^\dagger {{\varvec{\xi }}}^{\mu ,\lambda }$ is the adjoint (conjugate) vector of ${{\varvec{\xi }}}^{\mu ,\lambda }$, which satisfies the orthonormal relation $^\dagger {{\varvec{\xi }}}^{\alpha ,\rho }\cdot {{\varvec{\xi }}}^{\beta ,\sigma }= \delta _{\alpha \beta }\delta _{\rho \sigma }$ where $\delta$ is Kronecker’s $\delta$. We assume that the total number of attractor patterns is much less than the number of neurons, $KL\ll N$. When this condition was satisfied, and the connectivity parameter $r^{I(O)}_i$ approached full connectivity $r^{I(O)}_i=N$ for all *i*, then the cyclic sequences of patterns used to construct the synaptic connection matrix was stable cycle attractors. Therefore, the network functioned as an associative memory in the following way. If ${{\varvec{s}}}(t)$ is one of the memory (attractor) patterns ${{\varvec{\xi }}}^{\mu ,\lambda }$, then ${{\varvec{s}}}(t+1)$ will be the next memory pattern ${{\varvec{\xi }}}^{\mu ,\lambda +1}$ in the cycle. If ${{\varvec{s}}}(t)$ is near the memory pattern ${{\varvec{\xi }}}^{\mu ,\lambda }$ then the sequence ${{\varvec{s}}}(t + lL)~~(l=1,2,3,\cdots )$ generated by the *L*-step map converges quickly into the memory pattern ${{\varvec{\xi }}}^{\mu ,\lambda }$. Each memory pattern ${{\varvec{\xi }}}^{\mu ,\lambda }$ has a set of states $B_{\mu ,\lambda }$, called an attractor basin, such that if ${{\varvec{s}}}(t)$ is in $B_{\mu ,\lambda }$, then ${{\varvec{s}}}(t+lL)~(l=1,2,3,\cdots )$ converges into ${{\varvec{\xi }}}^{\mu ,\lambda }$.

On the other hand, if input connectivity $r^I_i$ for all *i* are reduced sufficiently by depletion of the synaptic link matrix, the attractors become unstable. Various aspects of the dynamics at reduced connectivity have been discussed in our previous studies about the network state ${{\varvec{s}}}(t)$. In particular, it has been found that in a regime of certain small $r^I_i$ for all *i*, chaotic wandering occurs repeatedly but aperiodically in all the areas of state space that were attractor basins at full connectivity. These extraordinary situations have been called “chaotic itinerancy”, based on the general viewpoint about the roles of chaos in biological information processing, including the brain^46–49^.

In the present study, we extended the basic formulation of our recurrent neural network, described above, to our three-module model. Let us introduce index $\kappa$ (or $\eta$) that indicates the visual module (*V*), memory module (*M*), and perceiving module (*P*). Therefore, state updating can be achieved with the use of Eq. (1) and Fig. 2

3 $$\begin{aligned} s^\kappa _{i}(t+1) = \mathrm{sgn} \left[ \sum _{\eta } \sum _{j=1}^{N_\eta } \varepsilon _{ij}^{(\kappa , \eta )} w^{(\kappa , \eta )}_{ij} s^\eta _{j}(t) + q_i \delta _{\kappa , V} - \theta ^\kappa _i \right] , \end{aligned}$$

where $\delta$ is Kronecker’s $\delta$, so $\{q_i\}$ exists only when $\kappa = V$ (Visual module), and represents external input signals that are set to be arbitrary strength applied to each neuron in visual module $s^V_i$. Additionally, $\{ w^{(\kappa , \eta )}_{ij} \} \; (\kappa (\eta ) = V, M, P)$ represents intramodule and/or intermodule interactions, respectively. $\{ \theta ^\kappa _i \}$ is the threshold of neurons belonging to each module; in our computer experiments, they play very important roles, as discussed in the previous section, which represents the regulatory effects of neuromodulators on the activity levels of entire networks.

**Figure 3:**
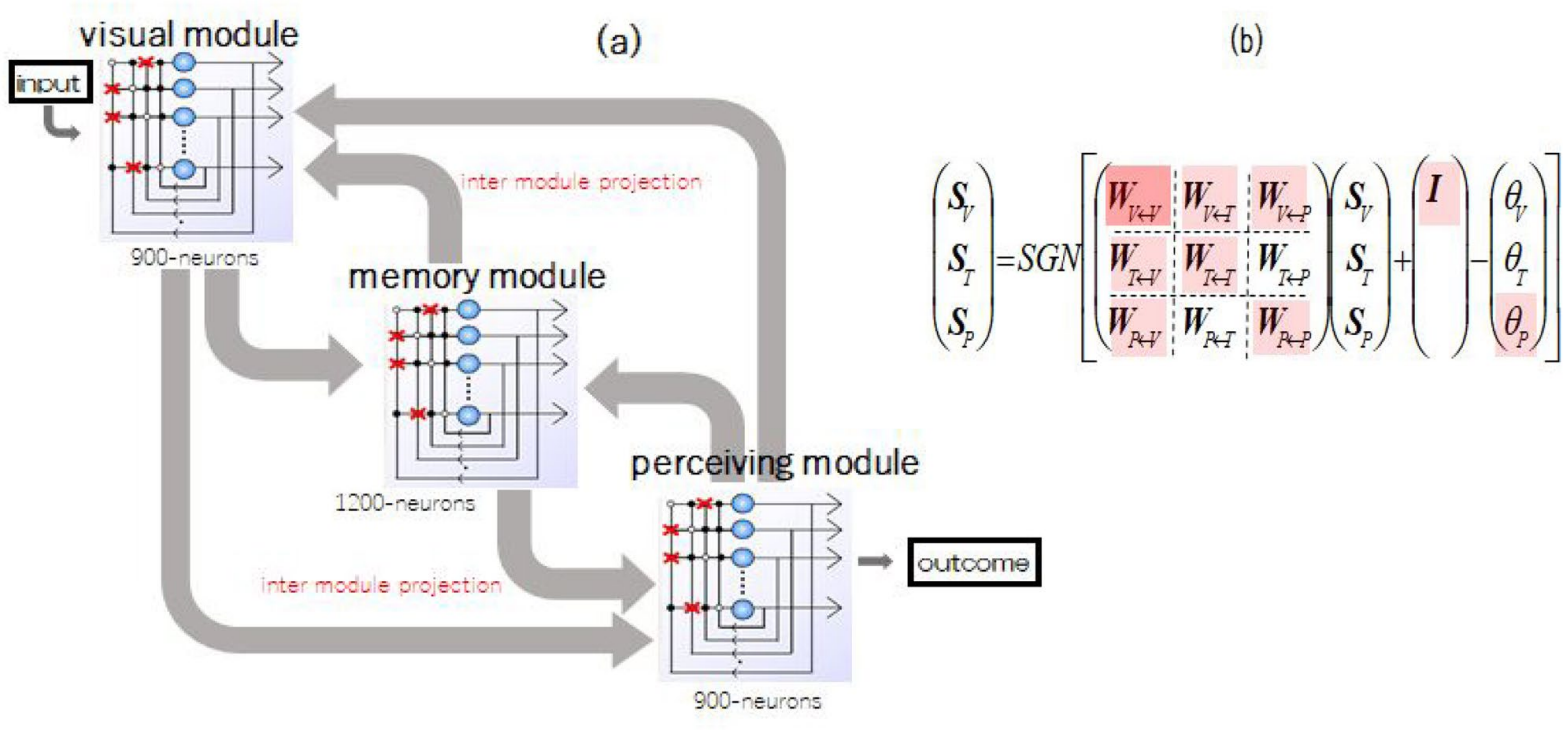
(**a**) Block diagram of our three-module model. (**b**) Symbolic representation of neuron updating, where the red crosses in (**a**) is schematical images of dysfunctional synaptic connections and the light red color in (**b**) indicate atrophy suggested based on the data obtained by inspection technologies, though this speculation is only qualitative. In our computer experiments shown in the text, the quantitative rate of necrosis in each red part is determined, as discussed in detail.

In the present experiments, the number of neurons in each module is taken to be $N_V=900, \; N_M=1200, \; N_P=900$. $\{ \varepsilon _{ij}^{(\kappa , \eta )} \}$ is a matrix used to categorize a neuron as dead and/or prune the intrasynaptic connections in all modules and all intermodule synaptic connections. At present, all elements of $\{ \varepsilon _{ij}^{(\kappa , \eta )} \}$ are taken to be unity, which corresponds to the absence of atrophy in the brain. Updating of all neurons and signal transfers in this three-module system is schematically shown in Fig. 3. Red crosses in (a) and thin red colors in (b) in Fig. 3 represent that if neurons in a certain module are affected by disease, then defects in neural elements and pruned connections corresponding to necrosis of neural cells and/or synaptic connections due to disease are schematically shown as in the figure. These aspects are introduced by setting selected elements of $\{ \varepsilon _{ij}^{(\kappa , \eta )} \}$ to null and will be discussed in detail later.

**Figure 4:**
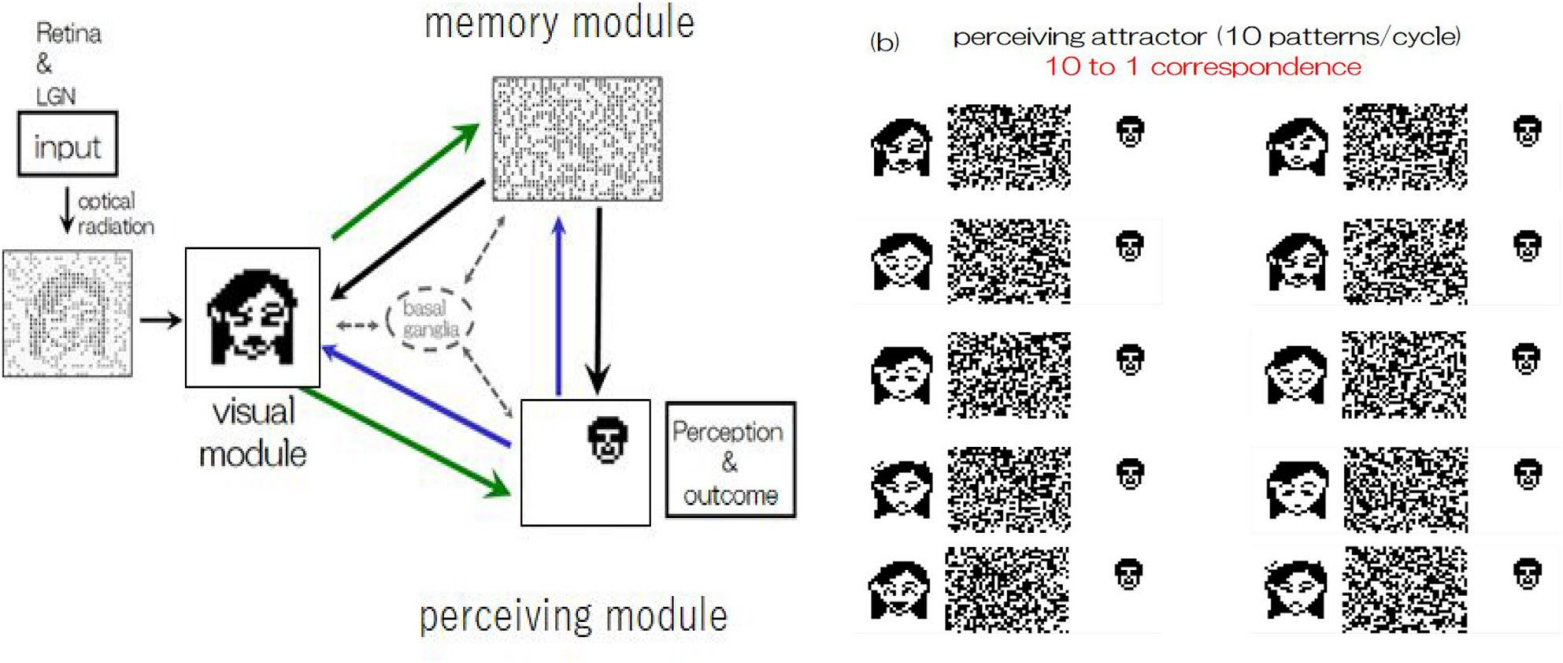
(**a**) Block diagram of a perceiving attractor in our three module model, where neuron activity is represented by bit patterns for convenience sake to visualize them. (**b**) One of the employed perceiving attractors, each of which consists of 10 periodic sequence of bit patterns (state vectors) in each module.

Now, we describe the derivation of the perceiving attractors that were employed in this experiment. As noted in the previous section, a total of 11 limit cycle attractors were embedded in this three-module system, where each limit cycle consisted of a periodic sequence of 10 patterns, as shown in Fig. 4. As a matter of convenience regarding visualization of neuron activity, the state vector in the visual module was represented as $30 \times 30$ bit pattern, $30 \times 40$ random bit pattern in the memory module and $30 \times 30$ bit pattern in the perceiving module. In state vector representation, we employed $\{ {{\varvec{\xi }}}^{\mu \lambda } \} \rightarrow \{ ( {{\varvec{\xi }}}^{\mu \lambda }_V, {{\varvec{\xi }}}^{\mu \lambda }_M, {{\varvec{\xi }}}^{\mu \lambda }_P ) \}$ as a set of perceiving attractors. Hence, the total number of components (total dimension) was 3, 000. Note that in the visual and memory modules, 10 patterns per cycle were embedded; however, in the perceiving module, one fixed pattern was embedded during updating to represent recognition perception with ten-to-one correspondence mapping (see Fig. 4), which is metaphor of a specific conceptual perception incorporated with many patterns in visual and memory modules.

**Figure 5:**
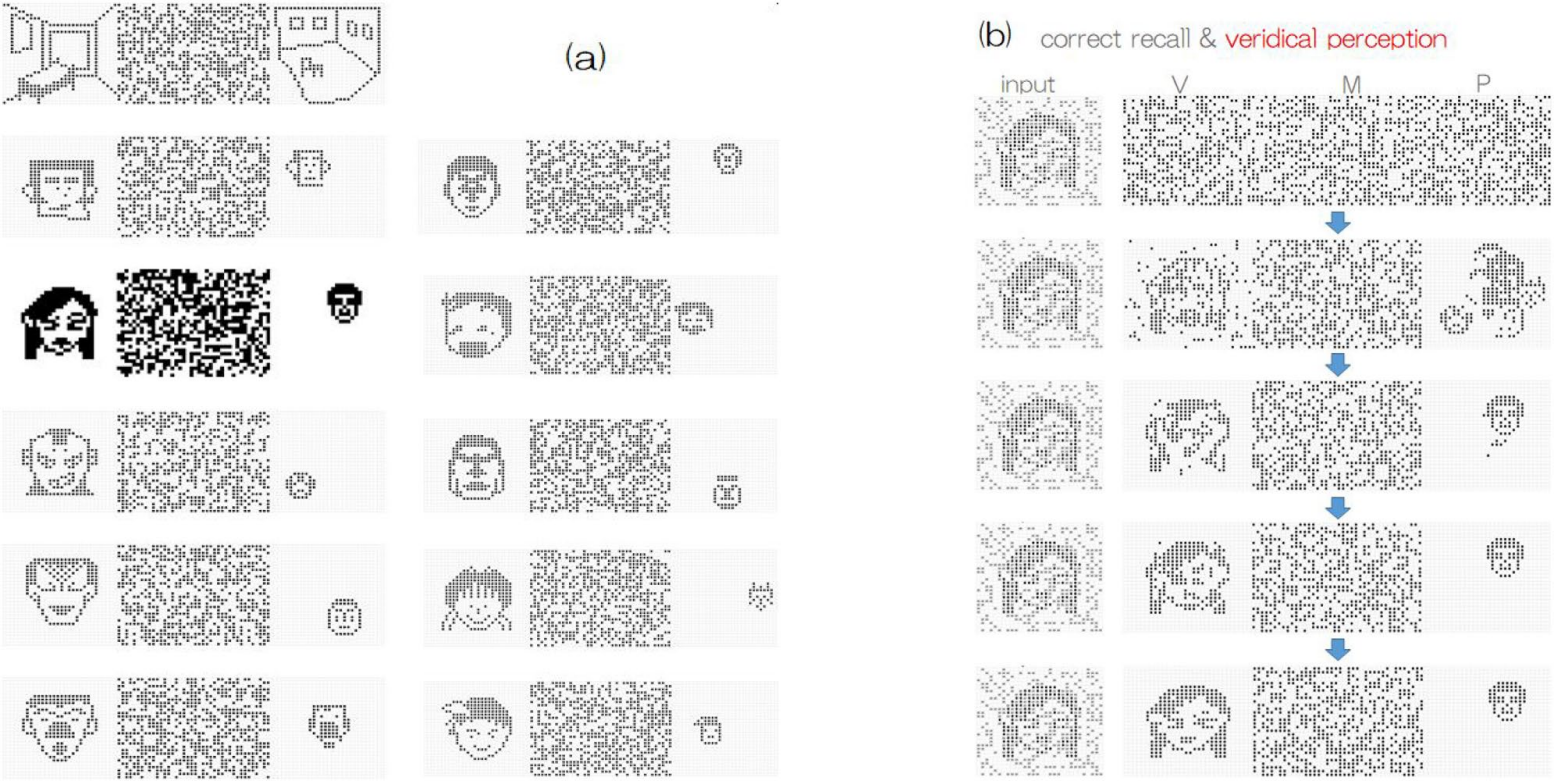
(**a**) Eleven perceiving attractors were used; where each attractor (limit cycle) contains 10 periodic bit-pattern sequences in each module. (**b**) An example of a converging update of neural activity from noisy inputs, which corresponds to veridical perception. Note that regardless of how noisy or deformed input deviating from the embedded 10 patterns is incorporated into the visual module, updating the activity gives the same fixed pattern in the perceiving module. This means that “ten to one correspondence mapping” was embedded in all perceiving attractors and is regarded as a metaphor of concept formation via veridical perceptions. In the Supplementary materials of this paper, readers are able to see animations of the time course in neural activity updating.

In Fig. 5, snapshots of the eleven attractors are shown, where each attractor consists of a periodic sequence of ten patterns, as shown in Fig. 4 (see Supplementary materials in which cyclic pattern animations with a 10-step period are shown). The right side in Fig. 5 shows the first several updating steps after noisy input is applied to the visual module. During state update following Eq. (3), state vectors in the three modules converge into the specific periodic sequence of embedded attractors that corresponds to veridical perception with respect to noisy input.

## Results

### Hallucinating-like destabilization of perceiving attractors due to reduction in characteristic neurons and synaptic connectivities

Our next task was to investigate the destabilization of perceiving attractors caused by various defects of neural elements and/or those in the synaptic connection matrix where they are regarded as areas of the neural network with irreversible but partial damage. In later descriptions, we present the results of computer experiments in detail. Here, we discuss the global damage depicted in Fig. 3 by red color, where the degree of degeneration or defect is not yet specifically shown but it should be roughly estimated based on the data, i.e., DTI-MR, MRI, etc., from patients with DLB. A computational representation of damage was created; for example, when the *Q*-th neural element is dead, then all corresponding row and column elements of $\varepsilon ^{(\kappa , \eta )}_{ij}$ are set to be null; that is, $\{ \varepsilon _{Qj}^{(\kappa , \eta )}=0, \; \varepsilon _{iQ}^{(\kappa , \eta )}=0 \; | \; i, j = 1 \sim N \;; \; \kappa (\eta )=V, M, P \; \}$. Impairment or dysfunction of synaptic connections are introduced by specific setting of $\{ \varepsilon _{ij}^{(\kappa , \eta )} \}$. In principle, it is possible to introduce dysfunction of presynaptic connectivity and postsynaptic connectivity independently; however, without loss of generality, we confined ourselves to only incoming (fan-in) connectivity in the present experiments for computational simplicity; that is, we employed connectivity parameters as $\sum _j \varepsilon ^{(\kappa , \eta )}_{ij}=r^{(\kappa , \eta )} \;\; \mathrm{for \; all}\;\; i \; \; (i = 1 \sim N_{\kappa (\eta )}), \; \; 0 \le r^{(\kappa , \eta )} \le N_{\kappa (\eta )}$ for living neuron elements. Note that the suffix of intra- and intermodule-projection in $(\kappa , \eta )$ means $\eta \rightarrow \kappa$ and it is same with suffixes in the matrix *W* in Fig. 3. Note that $r^{(\kappa , \eta )}$ is occasionally written as $r^{(\kappa \leftarrow \eta )}$ to show axonal projections explicitly.

As two characteristic examples of partial damage in the present system, we describe two exemplary cases below.

1. Destabilization due to “impairment of bottom-up information from the visual module” and simultaneously that from the memory module, where $r^{(P \leftarrow V)}+r^{(P \leftarrow M)}=1050$ with randomly selected configurations from 2100 synaptic connections.
2. Mildly modulated dysfunction of veridical perception due to “fluctuation of thresholds in the perceiving module corresponding to impairment in neuromodulator release”, where we employ $\theta _P = 0.02 \mathrm{sin} (2 \pi t_n/100).$

**Figure 6:**
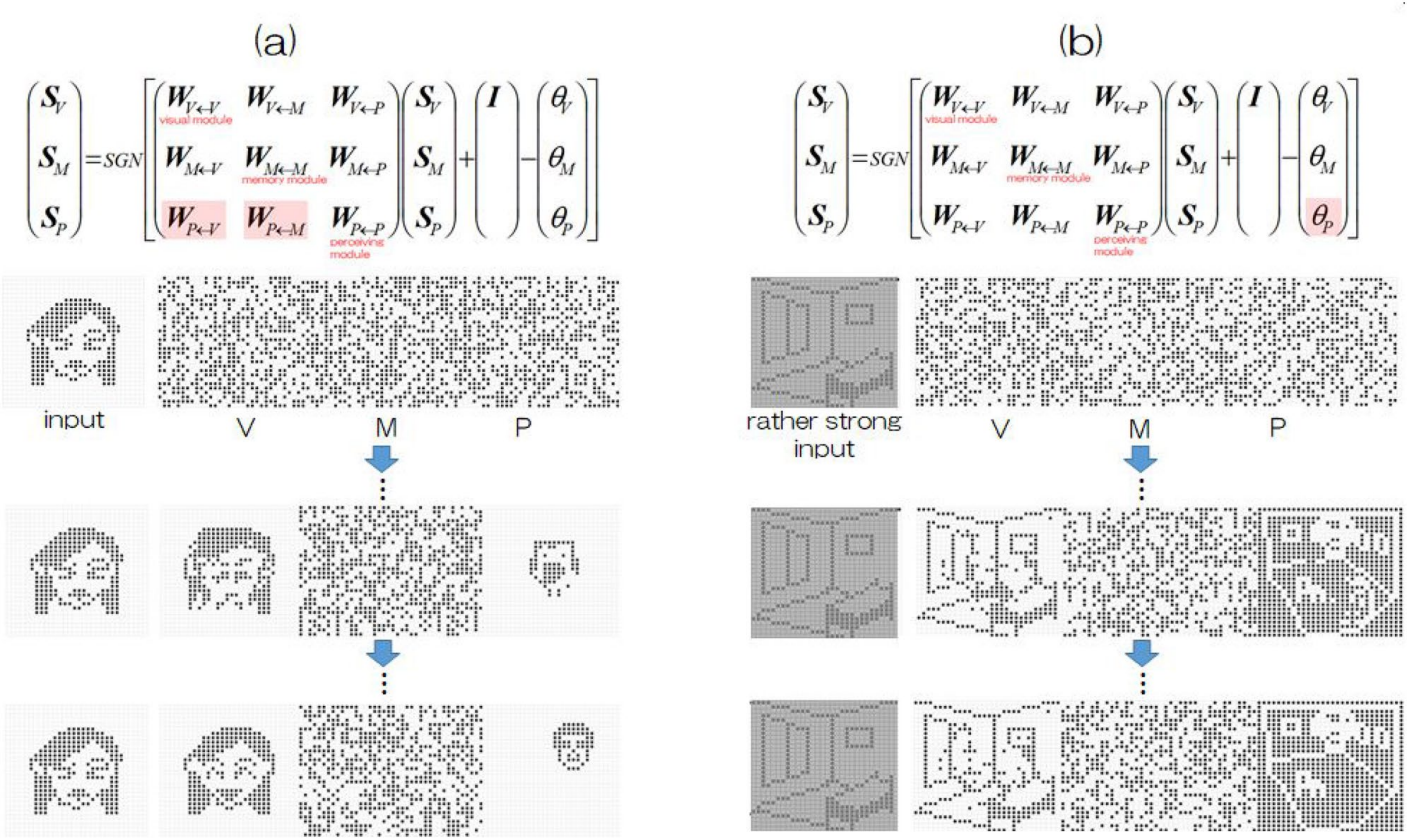
(**a**) An example of deficits in bottom-up information giving rise to erroneous perception initially but finally resulting in veridical perception. (**b**) When thresholds of the perceiving module fluctuate, even rather strong input leads to erroneous perception. Thus, if there are very slow variations in the fluctuations of neuromodulator release due to basal ganglia impairments, it may account for the appearance frequency of hallucinations in patients with RCVH-DLB, for instance, one time per day or a few times per week. In the Supplementary materials of this paper, readers are able to see animations of the time course in neural activity updating.

The former is shown in Fig. 6 (the left side) by a light red color in the synaptic connection matrix and the latter depicted is on the right side in the same way. In the former case, neural activity indicates erroneous perception initially but ultimately produces veridical perception. In the latter case, even rather strong input results in a mix with other perceptions depending on fluctuations of thresholds in the perceiving module, where in the Supplementary materials of this paper, readers are able to see the corresponding animations describing the time course of neural activity. Thus, this information suggests that very slowly varying impairments of neuromodulator release from the basal ganglia may account for the appearance frequency of hallucinations in patients with RCVH-DLB, for instance, one time per day or a few times per week.

Now, we introduce all the factors of cell death and dysfunction of synaptic connections in brain diseases that were considered; however, it should be noted that this model includes a large number of parameters, that is,

1. The number and configurations of dead neural elements in the three modules
2. The number and configurations of dysfunctional intra- and/or inter-module synaptic connections in the three modules, where it is possible to choose both or either presynaptic and/or postsynaptic connections

It is almost impossible to investigate all potential bifurcation phenomena in the destabilization of perceiving attractors due to a combinatorial explosion that would occur when selecting a large number of parameters even if we estimated them based on observed or measured data acquired by MRI, PET, DTI-MR, SPECT, etc. Moreover, one should be aware that all data have considerable uncertainty not only due to the individuality of patients but also due to the inaccuracy inherent in measurements, particularly the number of dead cells and configurations, the number of surviving synaptic connections and the configurations, acquired by noninvasive techniques.

**Figure 7:**
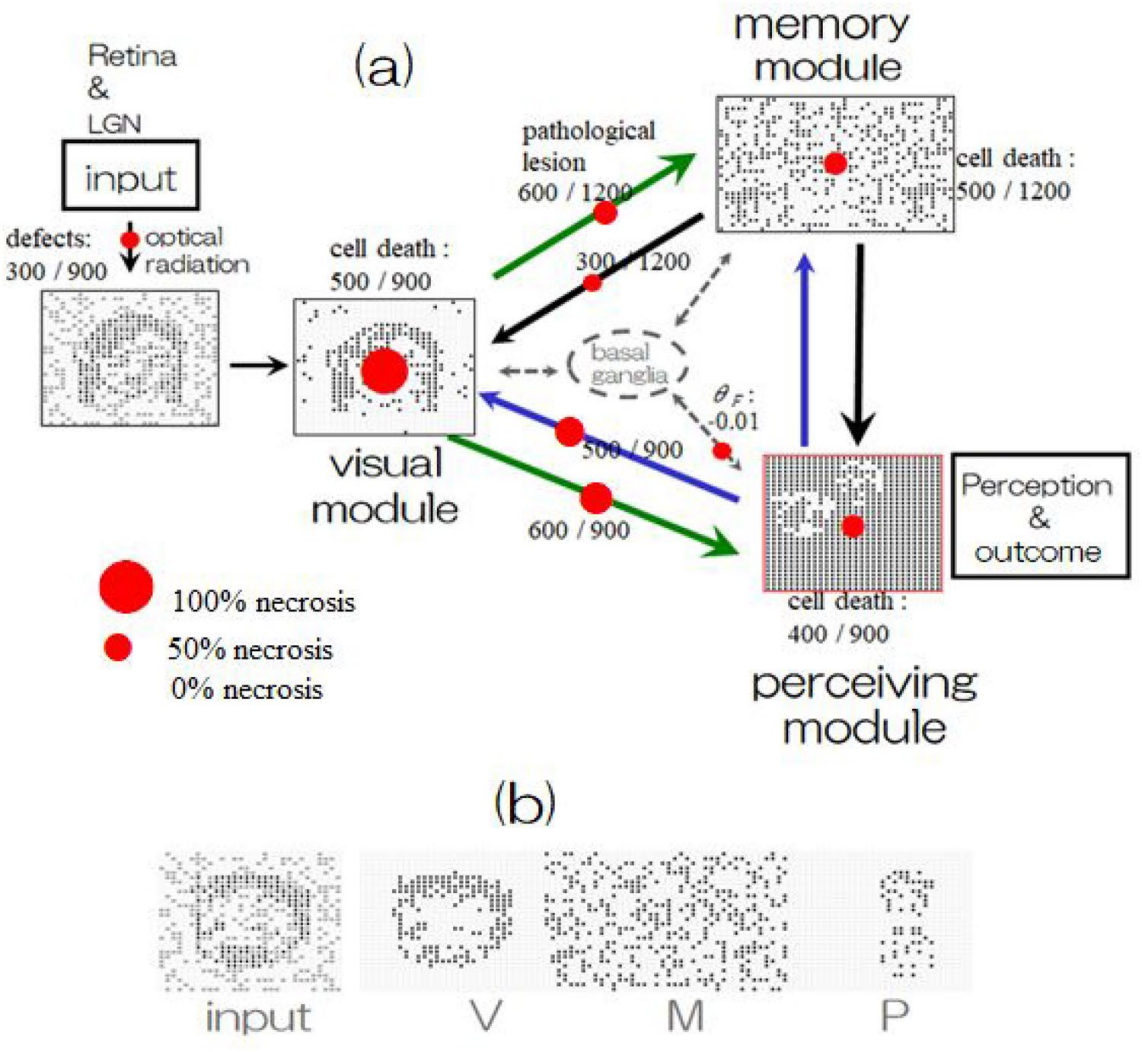
(**a**) Red colors in the block diagram of each module and association fibers (synaptic connection) shown by arrows indicate atrophy obtained by noninvasive measurement technologies, MRI, DTI-MR, etc., where the numbers (the rate of necrosis) were determined [after trial and error] of executing experiments around the speculated values suggested by measurements. The sizes of the red circles are proportional to the rate of necrosis in each module and connectivity, but only the red circle of the basal ganglia represents the threshold change due to necrosis. It should be noted that by noninvasive measurement, no atrophy was detected in the association fibers (uncinate fasciculus) between the temporal module and perceiving module; however, in the present model, synaptic connections were lacking due to cell death in the memory (temporal) module and perceiving (prefrontal) module, which were not indicated explicitly. Activity patterns show hallucination-like perception in response to noisy input into the visual module, where cell death numbers and degenerate numbers of synaptic connections are depicted in the figure. (**b**) Another example of hallucination-like perception for different noisy inputs. In the Supplementary materials of this paper, readers are able to see animations of the time course in neural activity updating.

Thus, it is unavoidable to rely on a “trial and error” approach even after selecting a certain set of parameter values, which means that we should try changing connectivity and configuration parameters around the chosen value set. In our experiments, for example, once specific amounts of dead cells belonging to one module were selected, then several options were executed, each of which has a different random configuration under the same necrosis number. When the results show similar phenomena with high probability in given number of trials, then they were regarded as “typical cases” occurring in the selected parameter set. It is possible to find many typical cases of hallucination-like instabilities by means of manageable trials in which parameters of cell death and the number of degenerated synaptic connections are assessed by executing them in different configurations. Two such typical cases are shown in Fig. 7. Both cases show hallucination-like erroneous perception but do not appear in the other parameter values as long as the values are *considerably different*. Thus, it should be noted that their occurrences are *not deterministic* but *quasi-deterministic*, as observed in complex systems, including the brain. Readers are able to see the corresponding animations describing the time course of neural activity in the Supplementary materials of this paper.

## Discussion and concluding remarks

**Figure 8:**
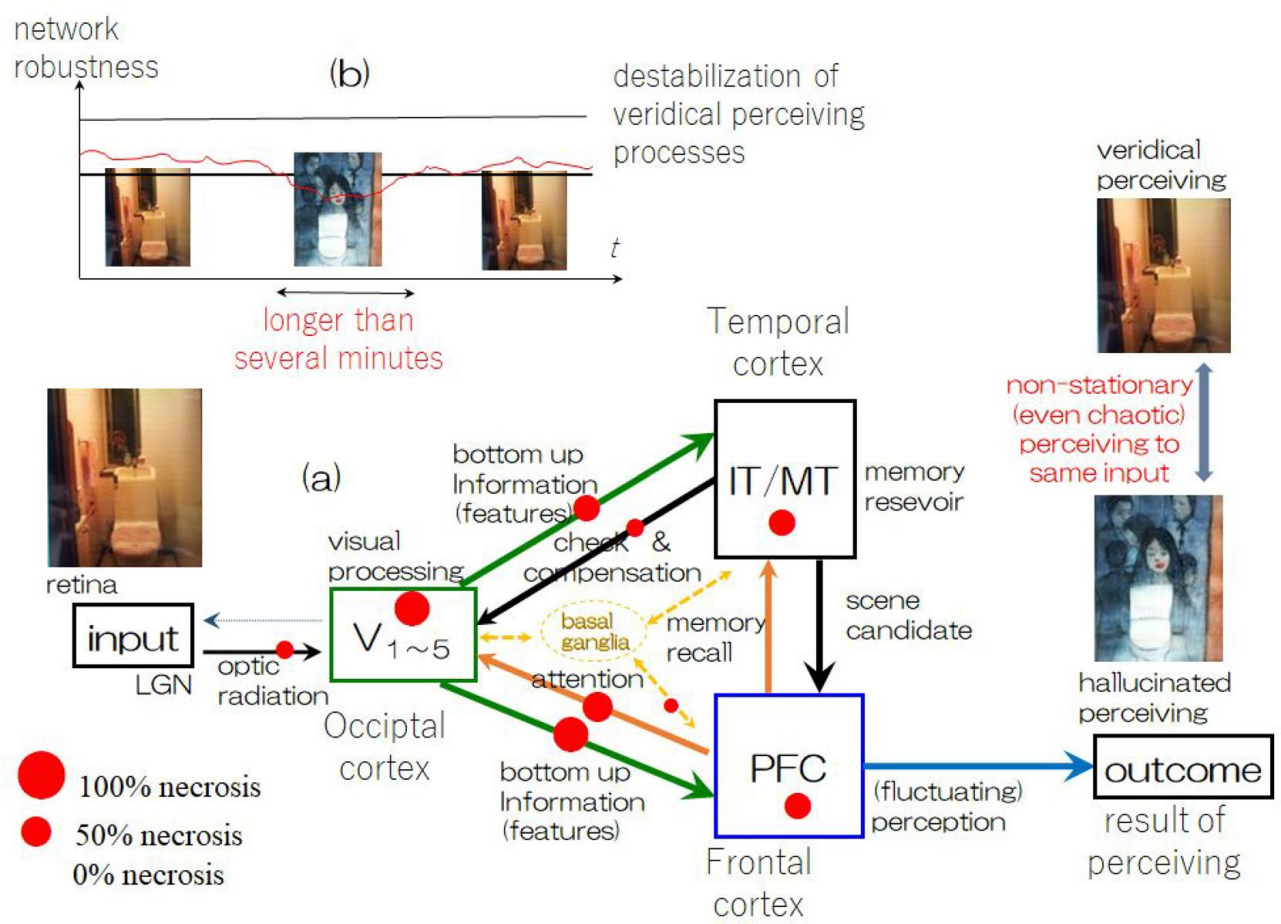
(**a**) Our concluding scenario of RCVH-DLB based on our investigation about of the pathological consideration mechanism of DLB and the computer experiments using our three-module model. Red colors in the block diagram indicate atrophy determined by computer experiments shown in Fig. 7. (**b**) Schematic figure of the transient appearance of the hallucinatory state expected by introducing certain fluctuations of in the thresholds of neurons in each module.

In the brain, veridical perception is supported and/or sustained by collective fields that exchange advanced information via interaction among many regions (lobes) while maintaining self-consistency. A small number of defective cells and/or synaptic connections do not severely affect collective fields; however, gradual accumulation of irreversible damage due to disease or aging, etc., will cause systems to approach edge of instability, which we call destabilization of perceiving attractors in the present case. Thus, by means of computer experiments in our drastically simplified model, we can see “metaphoric phenomena of RCVH” in complex bifurcation phenomena associated with destabilization of perceiving attractors in large dimensional state space of artificial neurons activity, as typical corresponding phenomena of RCVH-DLB summarized in Fig. 8. Here, we note that there are a considerable number of cases of hallucinations in which multiple patients in the same location report the same hallucination (D. Collerton et al. 2016). One perspective suggests that hallucinations could be induced by certain stationary bias resulting from specific deficits of neural functioning. However, it should be noted that there are also many cases in which the appearance of hallucinations is not as stationary as shown in the same reference and others. In particular, hallucinations of insects and/or animals tend to appear nonstationary. Therefore, from the perspective of pathological observations in neural systems, certain systematic defects do not always evoke specific hallucinations. Additionally, we have shown that such phenomena in our computer experiments should be described as *neither* “accidental occurrence” *nor* “deterministic occurrence”, because completely random cell death and random pruning of synaptic connections certainly does not destabilize perceiving attractors in a way that would cause hallucination-like bifurcations; moreover, we *are unable to state* that a specific set of parameters definitely creates a specific hallucination-like bifurcation structure in destabilizing situations.

These situations have been observed in many complex systems in nature, including biological systems, as critical phenomena (precursor phenomena) associated with phase transitions occurring in many-body (nonlinear) systems. It is generally accepted that, as one of the typical dynamic processes of destabilization, various kinds of fluctuations occur transiently but do not decay in short periods of time. These transient dynamics are unpredictable on a long time scale and are called “weak chaos or critical fluctuations”in the physics of phase transitions, particularly in systems that have finite but large degrees of freedom, such as biological systems.

If the occurrence of such weak chaos results from certain harnessing mechanisms, for instance, functional actions of inhibitory neurons in neural networks, then they are functionally useful and effective dynamics. Thus, these mechanisms can be used to create adaptive functions that are sensitive in their responses to changes in environments, i.e., a point that the authors have insisted upon^46–49^. However, when these results (instabilities) are due to irreversible necrosis of active elements caused by diseases or injuries, such weak chaos is regarded as a pathological symptom that indicates precursor phenomena of functional breakdown and make systems susceptible to network diseases.

Our computational model and computer experiments show that instabilities in this model network cause hallucination-like destabilization to occur in rather rare combinations of necrosis of characteristic neurons and synaptic connections in visual perception systems. However, correspondences between the data obtained by advanced measurement technologies and our computational model are far from an expected unified viewpoint of measurements and theoretical considerations. We must recognize that a detailed understanding of the mechanisms of RCVH-DLB remains obscured by the difficulty of complex hierarchical systems consisting of finite but large degrees of freedom that nonlinearly interact with each other.

## Supplementary Information

Supplementary Information.
