# Supplementary material for: Visual hallucinations in dementia with Lewy bodies originate from necrosis of characteristic neurons and connections in three-module perception model
Source: Sci Rep. 2022 Aug 19;12:14172. doi: 10.1038/s41598-022-18313-6 (PMC9391481; doi:10.1038/s41598-022-18313-6)
Supplement: Supplementary file 1 — Supplementary Information. [file 41598_2022_18313_MOESM1_ESM.pdf]

Supplementary of the paper: Visual Hallucinations in Dementia with Lewy Bodies Originate from Necrosis of Characteristic Neurons and Connections in Three-Module Perception Model

Shigetoshi Nara[1], Hiroshi Fujii[2], Hiromichi Tsukada[3], and Ichiro Tsuda[4]

[1]Graduate School of Natural Science & Technology, Okayama University, Okayama 700-8530, Japan
[2]Faculty of Information Science and Engineering, Kyoto Sangyo University, Kyoto 603-8555, Japan
[3]Center for Mathematical Science and Artificial Intelligence, Chubu University, Aichi 487-8501, Japan
[4]Chubu University Academy of Emerging Science/Center for Mathematical Sciences and Artificial Intelligence, Chubu University, Aichi 487-8501, Japan

ABSTRACT

The supplementary materials and the animations of neuron activities in our three-module model are shown.

The animation corresponding to Fig. 5 in the paper

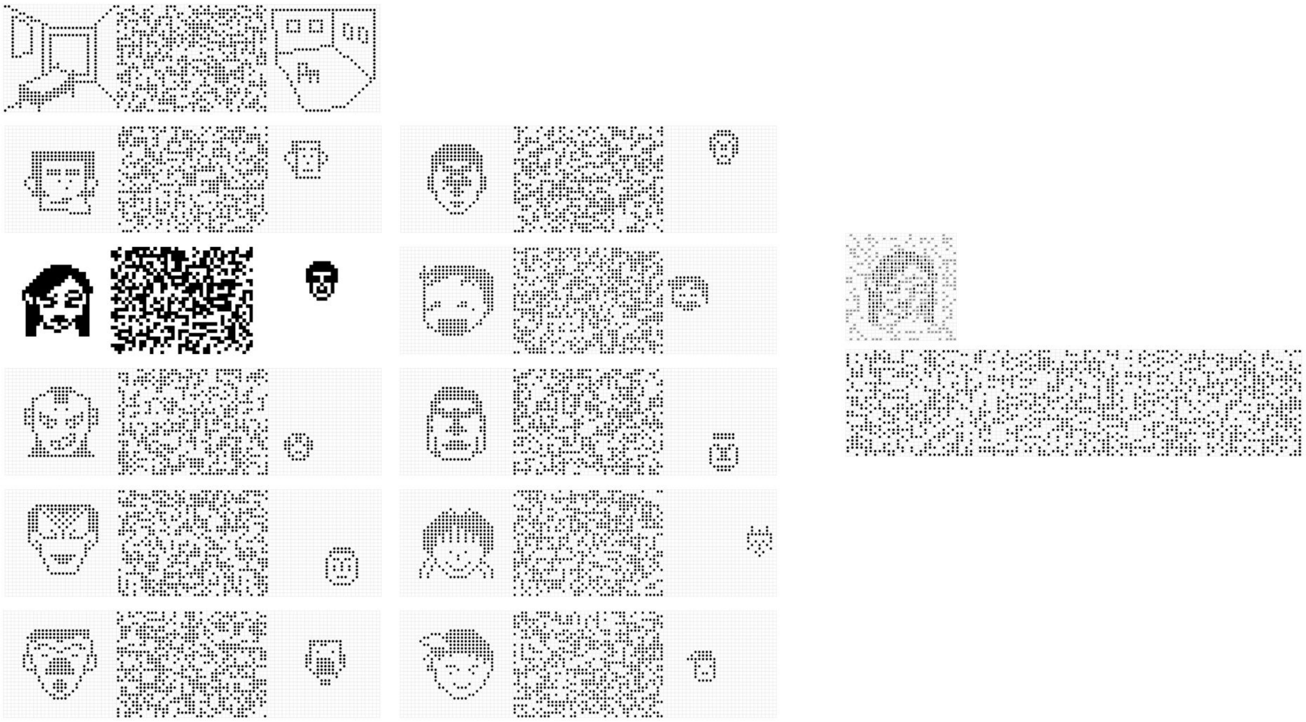

Let us note that the displaied sizes to show neuron-activity seem to be slightly different, but all are same pixel-number, visual module: 30×30, memory module: 30×40, perceiving module: 30×30 and, on the right side, noisy input to visual module: 30×30. The small difference of displaied sizes is generated during processes of image copy and paste from Exel-files.

The supplementary animation corresponding to Fig. 5 in the paper

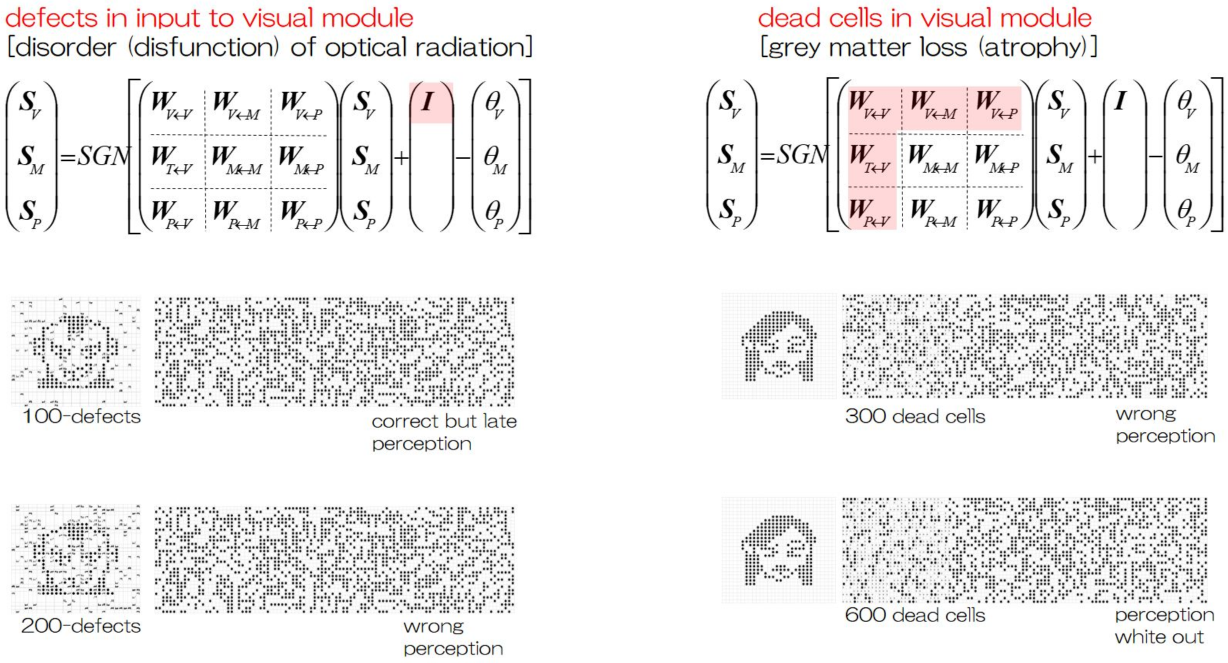

[Left]: An example that too many defects of input to visual module give wrong perception. [Right]: The other example that considerable dead cells in visual module bring wrong perception, particularly too many loss of cells results in complete loss of perception.

The animation corresponding to Fig. 6 in the paper

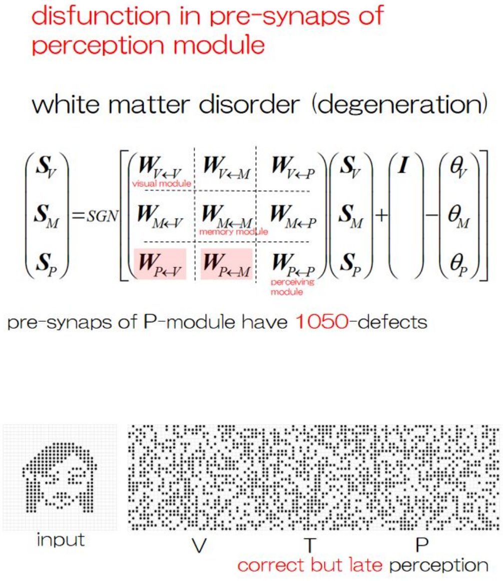

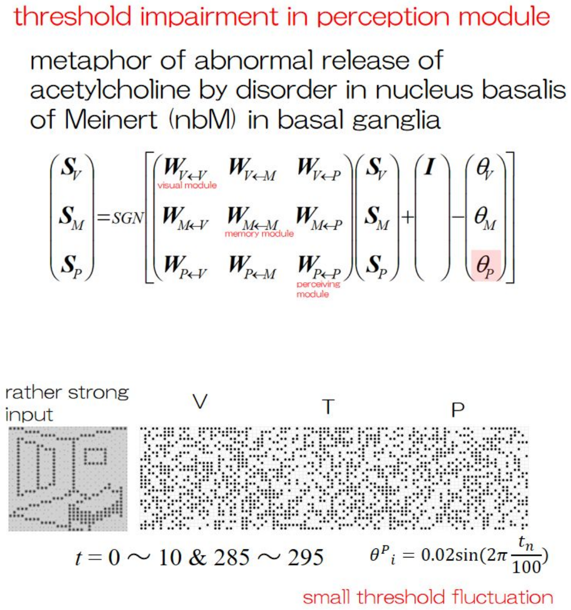

[Left]: An example that, with considerable deficits of *bottom up information*, neural activity indicates erroneous perception initially but finally settles down to a veridical perception. [Right]: Even rather strong input gives a mixing with the other perception depending on fluctuations of thresholds in perceiving module. Thus it suggests that, if very slowly varing fluctuation of neuro-modulator releasing by impairments of basal ganglia exists, it may account for appearance frequency of RCVH-DLB, for instances one time per day or a few times per week.

The animation corresponding to Fig. 7 in the paper

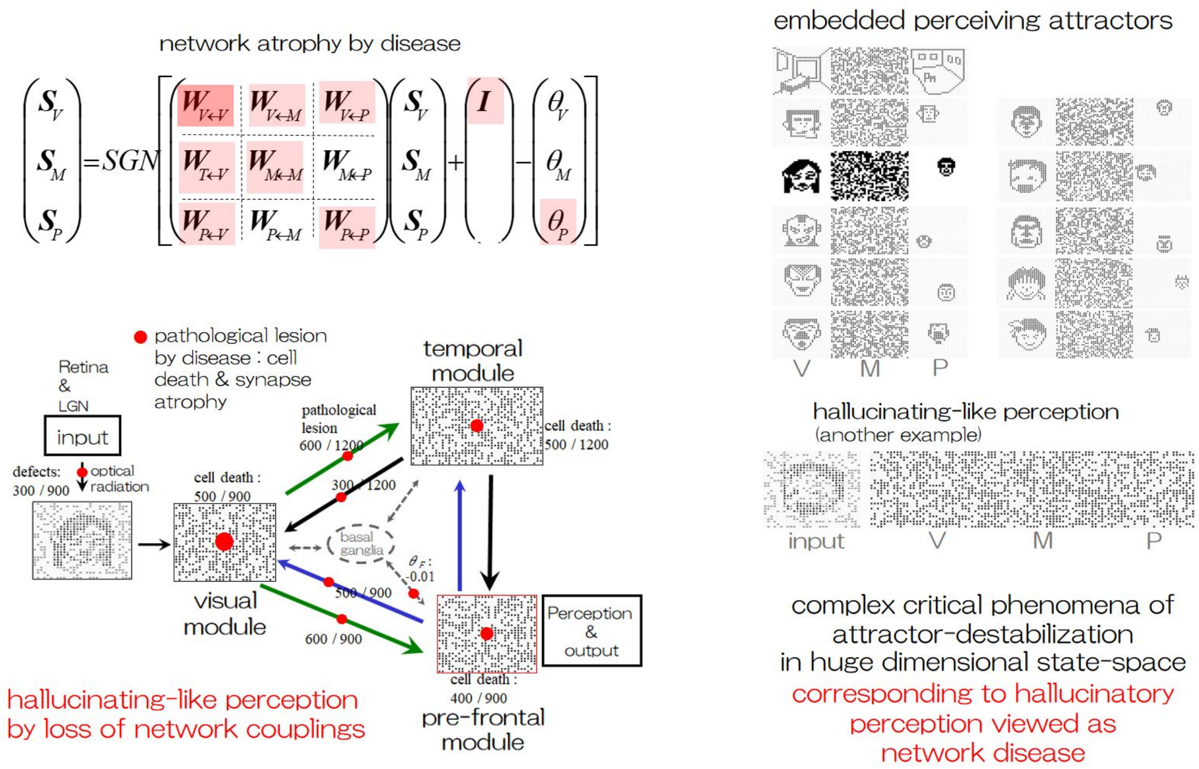

Two typical cases of hallucinating-like perception in our three-module model. Cell death numbers in each module and disfunctions of synaptic connectios are shown in the figure. Thin red colors in the block diagram of synaptic-connection matrix $W_{\kappa\leftarrow\eta}$ mean atrophy obtained by non-invasive measurement technologies, MRI, DTI-MR, etc., but the numbers of them are determined after [trial and error] arrond the speculated values given by measurements. It should be noted that, by non-invasive measurement, $W_{M\leftarrow P}$, and $W_{P\leftarrow M}$ have no atrophy, however in the present model they contain lacks of synaptic-connections due to cell death in memory (temporal) module and perceiving (pre-frontal) module shown in the figure.
